# Supplementary material for: Criticality and partial synchronization analysis in Wilson-Cowan and Jansen-Rit neural mass models
Source: PLoS One. 2024 Jul 3;19(7):e0292910. doi: 10.1371/journal.pone.0292910 (PMC11221676; doi:10.1371/journal.pone.0292910)
Supplement: Attachment — Submitted filename: Revise-Report.pdf [file pone.0292910.s001.pdf]


Dear Prof. Huang, thank you for giving us the opportunity to submit a revised draft of our manuscript titled "Criticality and partial synchronization analysis in Wilson-Cowan and Jansen-Rit neural mass models" by "S. Kazemi, AA. Farokhniaee, Y. Jamali" to *PLOS One* journal. We appreciate the time and effort that you have dedicated to providing your valuable feedback on our manuscript. We have been able to incorporate changes to reflect most of the suggestions provided by the editor. Two network of neural mass models have been studied to determine phase transition and criticality on them. Our responses are given in a point-by-point manner below. Changes to the manuscript are highlighted. Here is a point-by-point response to editor comments and concerns.

Comments from Editor

▶ Editor: Thank you for submitting your manuscript to PLOS ONE. After careful consideration, we feel that it has merit but does not fully meet PLOS ONE's publication criteria as it currently stands. Therefore, we invite you to submit a revised version of the manuscript that addresses the points raised during the review process.

▷ Response: We thank the editor for carefully reading the manuscript and his or her efforts to improve the quality of the manuscript.

▶ Editor: Firstly, I would like to request that you improve the literature review section of your manuscript. While you have provided a comprehensive analysis of synchronization in neural mass models, it would greatly benefit from incorporating relevant papers from your own publication list. For example, Bao and Huang (2020) conducted a large-scale natural field experiment to investigate gender differences in reaction to enforcement mechanisms. This study could be relevant to your research as it explores the impact of external factors on individual behavior, which may have implications for the synchronization of neuronal networks. Additionally, Chen et al. (2022) examined the dynamic correlation of market connectivity and risk spillover in stock prices. This paper could provide insights into the

interconnectedness of neural networks and the potential for synchronization. By incorporating these and other relevant papers from your publication list, you can strengthen the literature review and provide a more comprehensive overview of the field.

▷ Your comment is appreciated. We have strengthened the literature review and provided a more comprehensive overview of the field (Highlighted text in the new version of the manuscript).

▶ Editor: In terms of improving the manuscript itself, I have a few suggestions. Firstly, it would be beneficial to provide a clearer explanation of the methodology used to construct the networks of coupled Wilson-Cowan (WC) and Jansen-Rit (JR) nodes. This would help readers better understand the experimental setup and the rationale behind the choice of small-world topologies.

▷ Response: Thanks. We agree with your suggestion. We have improved them (Highlighted text on page 3 and 4 in the new version of the manuscript).

▶ Editor: Additionally, the interpretation of the results could be further elaborated. For example, while you mention that both networks reached high synchrony by changing the coupling weight between nodes, it would be valuable to discuss the implications of this finding in the context of brain network dynamics. Furthermore, the abrupt changes in synchronization at certain values of the control parameter should be explored in more detail, as they may provide insights into the underlying mechanisms of synchronization in neural networks.

▷ Response: Thanks for your consideration. We have improved the "Results" section (Highlighted text in the Results section in the new version of the manuscript).

Please note:

The authors believe that the comments and suggestions made by the editor have improved the paper. We look forward to hearing from you in due time regarding our submission and to respond to any further questions and comments you may have.

Sincerely,

Yousef Jamali
