# Supplementary material for: Criticality and partial synchronization analysis in Wilson-Cowan and Jansen-Rit neural mass models
Source: PLoS One. 2024 Jul 3;19(7):e0292910. doi: 10.1371/journal.pone.0292910 (PMC11221676; doi:10.1371/journal.pone.0292910)
Supplement: Attachment — Submitted filename: review paper (1).pdf [file pone.0292910.s003.pdf]

In agreement with the Editor, I believe the submitted work has notable merit. I appreciate that the authors have already made some steps following the Editor's suggestions, however, I believe major steps are still needed to reach publication quality. I outline my suggestions below.

This work is concerned with neural activity models on graphs. The manuscript would benefit from further discussing the difference, similarity, limitations, and novelty of the author's current work in comparison with other work in this field. For example:

Aqil, M., Atasoy, S., Kringelbach, M. L., & Hindriks, R. (2021). Graph neural fields: A framework for spatiotemporal dynamical models on the human connectome. *PLoS Computational Biology*, *17*(1), e1008310.

Conti, F., & Van Gorder, R. A. (2019). The role of network structure and time delay in a metapopulation Wilson--Cowan model. *Journal of theoretical biology*, *477*, 1-13.

Wilson, H. R. (2019). Hyperchaos in Wilson–Cowan oscillator circuits. *Journal of neurophysiology*, *122*(6), 2449-2457.

The discussion section should more clearly acknowledge the limitations of this work, and the extent of the claims of the authors. For example, it would be important to explicitly acknowledge that these results do not apply to the WC/JT models "in general", but to a specific formulation, with a specific parameter set, on a specific network topology. Different topologies, or different parameter sets, are likely to exhibit entirely different behaviors (see examples in the literature mentioned above). To be clear, I think it is entirely valid and warranted to focus on a single parameter set and a single topology, however the limitations of this approach need to be clearly stated. As has been often done in previous work, the authors manipulate the coupling strength as their main control parameter. It should be mentioned explicitly in the discussion that this analysis does not exclude that transitions could be generated also by varying other model parameters, or with different topologies.

As it stands, the methods section lacks clarity. I suggest that the authors work through the methods section to ensure that each step in the construction of their model can be reproduced. Choices should be justified based on biophysical reasons, or if they are pragmatic/arbitrary choices (i.e. choices taken "because it works") this should be clearly stated. Below, I outline some explicit suggestions.

The authors state that "Different initial conditions for both models are adjusted to show oscillatory activity in each independent run.". However, I am not able to find any further specifications or analysis on the role of the initial conditions, nor of the term P(t) in the WC model. It is not clear to me if the initial conditions are the same for all simulations or not, nor what these initial conditions are. The choice and role of specific initial conditions should be clarified explicitly and justified more clearly.

The authors provide an explanation of the classic DFA method. It remains unclear which frequency band DFA is applied in. As it stands, it appears to be calculated on the full signal. Generally, DFA is applied in specific frequency bands. Please clarify. I strongly recommend following closely the reference below:

Hardstone, R., Poil, S. S., Schiavone, G., Jansen, R., Nikulin, V. V., Mansvelder, H. D., & Linkenkaer-Hansen, K. (2012). Detrended fluctuation analysis: a scale-free view on neuronal oscillations. *Frontiers in physiology*, *3*, 450.

The authors explain that they construct their network topology as an (artificial) small world network with specific characteristics (K=20, p=0.2). The choice of small-world parameters K and p should be explained and justified further, since different (yet still small world) topology could potentially yield different results. If it is an arbitrary, pragmatic choice, this should be clearly stated in the discussion sections (limitations).

The authors also mention the use of DTI data, which has been used in other studies to construct realistic network topologies. I do not understand where DTI data comes in the construction of networks used in this work. My understanding is that the authors use 80 nodes connected in a small world topology. I do not see why the mentioning of DTI or brain atlases is warranted. To be clear, it is perfectly warranted and valid to study these models on an artificial small-world network. But this should not be confused with approaches using network topologies based on actual DTI or MRI data. If DTI data is used, make it more clear. If not, do not mention DTI data in your network construction section.

In the model formulation, it is not quite clear to me why the only coupling in the network is between excitatory populations (E-E). What is the reason not to have I-I, I-E, E-I coupling of different nodes? Please explain/justify this choice.

Looking at figure 2, it seems to me that, qualitatively, WC actually shows more complex/varied dynamics in the region of synchronization, with possibly some hint of traveling waves, and possibly different frequencies of oscillations; whereas JT shows a transition from completely desyncronized to completely sychronized oscillations, with seemingly constant frequency throughout the synchronous region. Some additional comments here could benefit the paper. Is the oscillation frequency of JT/WC constant throughout simulations, and throughout the synchronous zone, or does it vary?

The authors use the coefficient of variation (standard deviation over the mean, across simulations instances) as a “marker of criticality”. While I understand the rationale behind this choice, I would like to see better justifications or literature references detailing why CV over simulation instances would be accurate to detect criticality. For example, you could also also compute the CV *over time* for each simulation (standard deviation of the synchronization parameter over time), and then average that across simulations. This would give a measure of the variability of synchronization within simulation instances, which could also be an interesting metric and arguably also an apt marker of criticality.

Looking at the simulated activity, it is clear that synchronous oscillations in the WC model are much, much slower than in the JT model. The author’s chosen duration of 200s for the

simulation is then apt to capture many cycles of JT oscillations, but very few cycles for the WC model. Please consider running longer simulations for the WC model so that the number of cycles is at least comparable to JT, or adapt the WC model so that its temporal oscillations are closer in frequency to JT, so that the computation of their synchronization parameters relies on a similar number of cycles.

Consider running additional simulations for WC model for values of the control parameter *near* the expected transition points (e.g. try values 0.0625, 0.1875 and nearby). Your steps of coupling strengths are quite large for the WC model, and might be stepping over a transition.

Some figures lack clear axis labels. While the authors do provide information in the text and captions that clarifies the figures content, the figures themselves should also be readily understandable. For example, the x-axis of simulation figures should directly show the temporal duration; the units and significance of the DFA figure axes should not (only) use mathematical symbols, but also an explicit description (e.g. time window (seconds)).

I do not find any links to github or similar repositories for access to the code used in this work. Please provide links to static repositories of your code upon revision.
