# Supplementary material for: Criticality and partial synchronization analysis in Wilson-Cowan and Jansen-Rit neural mass models
Source: PLoS One. 2024 Jul 3;19(7):e0292910. doi: 10.1371/journal.pone.0292910 (PMC11221676; doi:10.1371/journal.pone.0292910)
Supplement: Attachment — Submitted filename: Revise-Report_PlosOne.pdf [file pone.0292910.s004.pdf]

Dear Prof. Cymbalyuk, thank you for giving us the opportunity to submit a revised draft of our manuscript titled ”Criticality and partial synchronization analysis in Wilson-Cowan and Jansen-Rit neural mass models” by ”S. Kazemi, AA. Farokhniaee and Y. Jamali” to *PLOS ONE* journal. We appreciate the time and effort that you and the reviewers have dedicated to providing your valuable feedback on our manuscript. We are grateful to the reviewers for their insightful comments on our paper. We have been able to incorporate changes to reflect most of the suggestions provided by the reviewers. Our responses are given in a point-by-point manner below. Changes to the manuscript are highlighted. Here is a point-by-point response to the reviewers' comments and concerns.

Comments from Editor:

▶ Editor: Please, edit the manuscript for clarity as suggested by the reviewers. The model needs further justification. Update the introduction and discussion with current references as suggested by the reviewer.

▷ Response: Thanks. We have done the reviewer's suggestions.

▶ Editor: Please, appropriately introduce “power-law behavior”, “ Wats-Strogatz topology”, and “the probability of rewiring” with appropriate references provided.

▷ Response: Thanks. The appropriate references have been added.

▶ Editor: Please, clarify, Was the model simulated only for one set of parameters?

▷ Response: Thanks for pointing this out. We have added these limitations in the ”Discussion and Conclusion” section: It is notable that different parameter sets and different topologies have not been discussed, which implies the limitation of our research.

▶ Editor: The notation for the JR system is not optimal as the choice of the same letter for the firing rate and the potential (line 114) impedes it comprehension. Furthermore, it's not clear why the difference $y_1 - y_2$ represents output of the model and postsynaptic membrane potential of pyramidal neurons, whereas y_0 was attributed to these neurons. The variables in Eq (4) are listed not in a logical order.

▷ Response: Thanks. The relationship between the incoming firing rate of pyramidal cells and the EEG signal is closely tied to the variable $y_1 - y_2$. The apical dendrites of pyramidal neurons are responsible for transmitting their postsynaptic potentials to the superficial layer of the cortex, which significantly contributes to the EEG signal (highlighted text in the "Jansen-Rit model" subsection). Moreover, we have provided a further explanation of the logical order in Eq(4) in our response to reviewer 3.

▶ Editor: Parameters in Table 1 for WC model are derived from a previous work of the authors but no information is provided for parameters in Table 2 for JR model.

▷ Response: Thanks. We have provided a further explanation of the information for parameters in Table 2 for JR model in our response to reviewer 1.

▶ Editor: In "Measure of criticality" section, the context is well introduced. I would add some examples regarding the assessed measures of criticality mentioned in line 169.

▷ Response: Thanks. We have provided a further explanation of some examples regarding the assessed measures of criticality in our response to reviewer 1.

▶ Editor: Why are simulations run 20 times? And in the 20 repetitions for each simulation, which are the changing parameters? Add an explanation about how the coupling strength range was set up for the simulations.

▷ Response: Thanks for your attention. The initial conditions change during repetitions, which can enhance precision and help mitigate bias in measurements. Repeated measurements are crucial for improving both the accuracy and precision of results. Precision refers to the consistency of results, and

by taking multiple measurements, the variability can be reduced, thereby increasing precision. This reduction in variability leads to more accurate results because the average of these values can reduce the overall error. The number of repeated simulations needed to increase accuracy depends on the model size and complexity. First, the simulations ran 10 times and we incresed them to 15 and 20 times. The results have not been changed during this process. So, we considered 20 times.

For explanation about the coupling strength range: First, a network with no copupling is considered. Each node shows a neural mass with Wilson-Cowan (Jansen–Rit) dynamics. Then, we incresed copuplig coefficient until each node leaves the limit cycle and rests in a fixed point (It has been added -highlighted text- in the 'Results' section).

► Editor: Change the title of heatmaps in figure 3 and 4 replacing alpha with K to be consistent with the rest of the manuscript.

▷ Response: Thanks for your comment. These figures have been edited.

Comments from Reviewer 1:

► Reviewer: The paper is well written and report the results of the comparison between JR and WC model in a concise way. The evaluation of computational model performance has a crucial importance in the field of computational neuroscience. For what concerning Editor's comments, in my opinion, the authors update the manuscript in a proper manner. Here below I share my comments concerning minor modifications which could improve the manuscript clarity.

▷ Response: We thank the referee for carefully reading the manuscript and his or her efforts to improve the quality of the manuscript.

► Reviewer: In the introduction the background is explain in a clear and concise form. However, some point it lacks practical examples, such as in line 37 and in line 41. I appreciated the intention to make an extensive overview of mean field applications in different fields, but I would add a practical example on how these implications can be explored by analyzing the mean field synchronization.

▷ Response: Your comment is appreciated. The following references have been added in the new version of the manuscript (highlighted text in page 2).

- Bocaccio H, Pallavicini C, Castro MN, Sánchez SM, De Pino G, Laufs H, Villarreal MF, Tagliazucchi E. The avalanche-like behaviour of large-scale haemodynamic activity from wakefulness to deep sleep. J R Soc Interface. 2019 Sep 27;16(158):20190262. doi: 10.1098/rsif.2019.0262. Epub 2019 Sep 11.
- Bick, C., Goodfellow, M., Laing, C. R., Martens, E. A. (2020). Understanding the dynamics of biological and neural oscillator networks through exact mean-field reductions: a review. The Journal of Mathematical Neuroscience, 10(1), 9.
- Scarpetta S, Morisi N, Mutti C, Azzi N, Trippi I, Ciliento R, Apicella I, Messuti G, Angiolelli M, Lombardi F, Parrino L, Vaudano AE. Criticality of neuronal avalanches in human sleep and their relationship with sleep macro- and micro-architecture. iScience. 2023 Sep 9;26(10):107840. doi:10.1016/j.isci.2023.107840.
- Wendling F, Koksal-Ersoz E, Al-Harrach M, Yochum M, Merlet I, Rufini G, Bartolomei F, Benquet P. Multiscale neuro-inspired models for interpretation of EEG signals in patients with epilepsy. Clin Neurophysiol. 2024 Mar 16;161:198-210. doi: 10.1016/j.clinph.2024.03.006. Epub ahead of print.

▶ Reviewer: All the parameters are explicit indicated in the description of WC equation but I suggest also adding the unit of measurement of each parameter to make equation clearer. Regarding the choice of using excitatory connection, this is true in the cerebrum, but in the cerebellum the connection between cerebellar cortex and deep cerebellar nuclei are driven by inhibitory coupling, therefore I suggest changing the sentence "as is true of the brain". As far as I understood, parameters in Table 1 for WC model are derived from a previous work of the authors but no information are provided for parameters in Table 2 for JR model. I recommend adding the reference if authors taken them from literature, or explaining how they are derived in this work.

The method used to build up the network is clearly reported. I would like to request to disentangle the meaning of adjacency and coupling parameters and how they are determined. Are they derived from the structural connectivity mentioned in the manuscript (lines 117-120)?

Regarding the network structure I would suggest adding some further details about what small-world topologies are. Furthermore, I recommend adding a comment about the reason why p is the most challenging parameters to be estimated.

In "Measure of criticality" section, the context is well introduced. I would add some examples regarding the assessed measures of criticality mentioned in line 169.

In "Simulations" section, simulations set up doesn't result completely clear to me. Why are simulations run 20 times? And in the 20 repetitions for each simulation, which are the changing parameters?

Furthermore, I recommend adding an explanation about how the coupling strength range was set up for the simulations.

▷ Response: Thank you for pointing these out.

- The unit of measurement of Time constant for excitatory (inhibitory) populations is s^{-1}.
- Thanks. We apologize for this error. We have removed "as is true of the brain" in the new version of the manuscript.
- According to Van Rotterdam's work, the parameters A, B, a, and b have been determined so that alpha activity can be produced by the system while respecting some basic properties of real post-synaptic potentials [9]. v_0, v_{max}, and r $= 0.56mV^{-1}$ are derived from the experimental studies of Freeman [2]. $C_i(i = 1, ...4)$ are proportional to the average number of synapses between populations.

Jansen and Rit reduced these quantities to a single parameter C based on a neuroanatomical study [3] that used synaptic counting to estimate these quantities:

$$C_1 = C$$
$$C_2 = 0.8C$$
$$C_3 = 0.25C$$
$$C_4 = 0.25C$$

Using these values, Jansen and Rit varied C to observe alpha-like activity and obtained it for C = 135 [5].

These explanations of JR model is highlited in the new version of the manuscript (page 4).

- Several statements that we made were more ambiguous than intended. So, we have explained more detail about network structure and meaning of adjacency matrix in the ”Network Structure” section to be more clearer. The matrix-based representation is a straightforward yet powerful method for illustrating the connectivity within brain networks. It provides a clear and concise way to map out the relationships between different neural components, facilitating a deeper understanding of the brain’s structure. This method is particularly useful in the context of network neuroscience, a rapidly evolving field that applies graph theory and other mathematical tools to study the complex systems. A fundamental tool in matrix representation is the adjacency matrix, denoted as M, shows connections between nodes. Specifically, $M_{ij} = 1$ indicates a connection exists between node i and node j, whereas $M_{ij} = 0$ signifies no connection.

  Here, this adjacency matrix is generated by the structre of WS network.
- The rewiring parameter (p) plays a pivotal role in determining the network’s small-world properties, as it influences the degree of randomness introduced into the network. At $p = 0$, the network is a regular lattice, while at $p = 1$, it becomes a random graph. $0 < p < 1$ result in networks that exhibit small-world characteristics, combining the benefits of both the lattice and random graph models. This flexibility allows for the exploration of how the small-world effect can be tuned by adjusting the rewiring probability, offering a powerful tool for studying the impact of network structure on various phenomena, from social networks to brain connectivity (highlighted text in the ”Network structure” section in the new version of manuscript).

- Power-law scaling of neuronal avalanches [1], Branching ratio [4], operating at the edge of a phase transition [6], and medium mean with high variability in synchrony value [8] are some hallmarks of criticality that have been observed in neural models (highlighted text in the "Measure of criticality" section).
- The initial conditions change during repetitions, which can enhance precision and help mitigate bias in measurements. Repeated measurements are crucial for improving both the accuracy and precision of results. Precision refers to the consistency of results, and by taking multiple measurements, the variability can be reduced, thereby increasing precision. This reduction in variability leads to more accurate results because the average of these values can reduce the overall error. The number of repeated simulations needed to increase accuracy depends on the model size and complexity. First, the simulations ran 10 times and we incresed them to 15 and 20 times. The results have not been changed during this process. So, we considered 20 times.

  For explanation about the coupling strength range: First, a network with no copupling is considered. Each node shows a neural mass with Wilson-Cowan (Jansen–Rit) dynamics. Then, we incresed copuplig coeficient until each node leaves the limit cycle and rests in a fixed point (It has been added -highlighted text- in the 'Results' section).

▶ Reviewer: Results are promising and prompt for applying SOPT analysis in brain modeling fields. Sometimes the authors referred the coupling parameter as "coupling parameter" or "K". This is a bit confusing, and I would suggest using "K" since it was already defined in methods part. Furthermore, I recommend changing the title of heatmaps in figure 3 and 4 replacing alpha with K to be consistent with the rest of the manuscript.

▷ Response: Thanks for your comment. These figures have been edited.

▶ Reviewer: Discussions are presented in a concise way. I would suggest adding the authors point of view supported by the manuscript results on when JR is more suitable than WC for brain dynamics modeling and the vice versa.

▷ Response: Thanks for your comment. In summary, neural mass models for modeling brain dynamics depend on several factors, including the specific characteristics of the neural activity being

modeled, the level of detail required, and the computational resources available. Both models have their strengths and are suited to different scenarios. JR model is more suitable for detailed neural dynamics and it provides a comprehensive framework for understanding the complex interactions within neural populations. Conversely, the WC focuses on the interaction between excitatory and inhibitory populations, providing a more straightforward representation of neural dynamics. While it may not capture the full complexity of neural interactions as the JR does, it is better suited for broader applications where a simplified yet accurate representation of neural dynamics is sufficient. The choice between these models should be guided by the specific requirements of the modeling task, including the level of detail needed, the presence of stochastic processes, and computational constraints (highlighted text in the ”Discussion and Conclusion” section).

Specific Comments:

▶ Reviewer: Line 54: I would replace “optimal” with “physiological”.

▷ Response: Agree. We have edited this word.

▶ Reviewer: Line 77, as many mean field approximations exists (such as Lorentzian base, heuristic TF based....), I would add a reference for the mean filed rational applied to WC model.

▷ Response: Agree. This following reference has been added:

Breakspear, M., 2017. Dynamic models of large-scale brain activity. Nature neuroscience, 20(3), pp.340-352.

▶ Reviewer: Line 132, if the “connections” are referred to structural connectome of neurons/brain regions, I would suggest using “biological” instead of physical.

▷ Response: Agree. We have edited this word.

▶ Reviewer: Line 137 The Reference for Watts-Strogatz topology is missed.

▷ Response: Agree. This following reference has been added:

Budrikis, Z. 25 years of small-world network theory. Nat Rev Phys 5, 440 (2023).

▶ Reviewer: Line 180: "long-range temporal correlations" is already defined as LRTC; therefore I would replace it with the acronym.

▷ Response: Agree. We have edited this word.

▶ Reviewer: Line 314 typo: Delete the "."

▷ Response: Agree. We have deleted ".".

▶ Reviewer: Figure 1: I suggest adding the title to the panels also in the figure to make it more clear at a first look. I would swap Panel A and Panel B consistently to the following figures where A is JR and B WC.

▷ Response: Thanks for your careful attention. The figure 1 have been edited (panel A shows JR and panel B shows WC model).

▶ Reviewer: Figure 4. Put [Hz] next to Rate as Activity[mV] in figure 3.

▷ Response: Thanks. We have edited these figures.

Comments from Reviewer 2:

▶ Reviewer: In agreement with the Editor, I believe the submitted work has notable merit. I appreciate that the authors have already made some steps following the Editor's suggestions, however, I believe major steps are still needed to reach publication quality. I outline my suggestions below.

▷ Response: We thank the referee for carefully reading the manuscript and his or her efforts to improve the quality of the manuscript.

▶ Reviewer: This work is concerned with neural activity models on graphs. The manuscript would benefit from further discussing the difference, similarity, limitations, and novelty of the author's current work in comparison with other work in this field. For example:

Aqil, M., Atasoy, S., Kringelbach, M. L., Hindriks, R. (2021). Graph neural fields: A framework for spatiotemporal dynamical models on the human connectome. PLoS Computational Biology, 17(1), e1008310.

Conti, F., Van Gorder, R. A. (2019). The role of network structure and time delay in a metapopulation Wilson–Cowan model. Journal of theoretical biology, 477, 1-13.

Wilson, H. R. (2019). Hyperchaos in Wilson–Cowan oscillator circuits. Journal of neurophysiology, 122(6), 2449-2457.

The discussion section should more clearly acknowledge the limitations of this work, and the extent of the claims of the authors. For example, it would be important to explicitly acknowledge that these results do not apply to the WC/JT models "in general", but to a specific formulation, with a specific parameter set, on a specific network topology. Different topologies, or different parameter sets, are likely to exhibit entirely different behaviors (see examples in the literature mentioned above). To be clear, I think it is entirely valid and warranted to focus on a single parameter set and a single topology, however the limitations of this approach need to be clearly stated. As has been often done in previous work, the authors manipulate the coupling strength as their main control parameter. It should be mentioned explicitly in the discussion that this analysis does not exclude that transitions could be generated also by varying other model parameters, or with different topologies.

▷ Response: Thanks for pointing these out.

- These references have been added (highlighted text in the third paragraph in the "Discussion and Conclusion" section).
- Your comment is really appreciated. We have added these limitations in the "Discussion and Conclusion" section: It is notable that different parameter sets and different topologies have not been discussed, which implies the limitation of our research. These results do not apply to the WC or JR models in general, but with a specific parameter set and on a specific network topology. Different topologies, or different parameter sets, are likely to exhibit entirely different behaviors. Moreover, we emphasis that these analysis does not exclude that transitions could be generated also by varying other model parameters, or with different topologies.

▶ Reviewer: As it stands, the methods section lacks clarity. I suggest that the authors work through the methods section to ensure that each step in the construction of their model can be reproduced. Choices should be justified based on biophysical reasons, or if they are pragmatic/arbitrary choices (i.e. choices taken "because it works") this should be clearly stated. Below, I outline some explicit suggestions.

The authors state that "Different initial conditions for both models are adjusted to show oscillatory activity in each independent run.". However, I am not able to find any further specifications or analysis on the role of the initial conditions, nor of the term P(t) in the WC model. It is not clear to me if the initial conditions are the same for all simulations or not, nor what these initial conditions are. The choice and role of specific initial conditions should be clarified explicitly and justified more clearly.

▷ Response: The authors are grateful for your detailed vision. The initial conditions refers to the initial value of varibles (E and I in WC model and $(y_0, ..., y_5)$ for JR model) and external inputs in both models. Theses initial conditions change during repetitions, which can enhance precision and help mitigate bias in measurements (highlighted text in the "Simulation" section in the new version of the manuscript). It is clearly obvious that for the same coupling coefficient, due to different initical conditions in each repeatation our result is dissimilar from another repeat, which shows the sensivity of the system to its initial conditions

In this paper, we consider the brain as a stochastic nonlinear dynamical system. It means that the external input for both models is defined as a stochastic parameter. Indeed, $p(t)$ is chosen randomly

from a Gaussian distribution (it has been added for more explanation of both models in the new version of the manuscript).

▶ Reviewer: The authors provide an explanation of the classic DFA method. It remains unclear which frequency band DFA is applied in. As it stands, it appears to be calculated on the full signal. Generally, DFA is applied in specific frequency bands. Please clarify. I strongly recommend following closely the reference below:

Hardstone, R., Poil, S. S., Schiavone, G., Jansen, R., Nikulin, V. V., Mansvelder, H. D., Linkenkaer-Hansen, K. (2012). Detrended fluctuation analysis: a scale-free view on neuronal oscillations. Frontiers in physiology, 3, 450.

▷ Response: Thanks for your careful considaration. Yes. Generally, DFA is applied in specific frequency bands. In experimental data, the received signal is composed of various frequencies. Therefore, it's necessary to separate these frequencies into different bands and then apply DFA technique to each band. In our research, we specifically set parameters from the two models that resulted in the signals showing only one frequency for each: the WC model exhibited delta frequency, and the JR model displayed alpha frequency. Consequently, we applied DFA to the full signal.

▶ Reviewer: The authors explain that they construct their network topology as an (artificial) small world network with specific characteristics (K=20, p=0.2). The choice of small-world parameters K and p should be explained and justified further, since different (yet still small world) topology could potentially yield different results. If it is an arbitrary, pragmatic choice, this should be clearly stated in the discussion sections (limitations).

▷ Response: Thanks for your comment. It is notable that different parameter sets and different topologies have not been discussed, which implies the limitation of our research. These results do not apply to the WC or JR models in general, but with a specific parameter set and on a specific network topology. Different topologies (changes in the number of nearest neighbors of a node or in the probability of rewiring), or different parameter sets, are likely to exhibit entirely different behaviors. Moreover, we emphasis that these analysis does not exclude that transitions could be generated also by

varying other model parameters, or with different topologies (it has been added in the ”Discussion and Conclusion” section).

▶ Reviewer: The authors also mention the use of DTI data, which has been used in other studies to construct realistic network topologies. I do not understand where DTI data comes in the construction of networks used in this work. My understanding is that the authors use 80 nodes connected in a small world topology. I do not see why the mentioning of DTI or brain atlases is warranted. To be clear, it is perfectly warranted and valid to study these models on an artificial small-world network. But this should not be confused with approaches using network topologies based on actual DTI or MRI data. If DTI data is used, make it more clear. If not, do not mention DTI data in your network construction section.

▷ Response: Thanks. We agree with this comment. Therefore, we have deleted the ”DTI data” in the new version of the manuscript.

▶ Reviewer: In the model formulation, it is not quite clear to me why the only coupling in the network is between excitatory populations ($E-E$). What is the reason not to have $I-I$, $I-E$, $E-I$ coupling of different nodes? Please explain/justify this choice.

▷ Response: Your comment is appreciated. Various methods exist for modeling the brain, including mesoscale or neural mass models. These models focus on interactions between distinct brain regions rather than individual neurons. Given that interactions between regions are primarily excitatory, with inhibitory interactions being localized, mesoscale models have been designed to account for these excitatory interactions exclusively [10].

▶ Reviewer: Looking at figure 2, it seems to me that, qualitatively, WC actually shows more complex/varied dynamics in the region of synchronization, with possibly some hint of traveling waves, and possibly different frequencies of oscillations; whereas JT shows a transition from completely desyncronized to completely sychronized oscillations, with seemingly constant frequency throughout the syn-

chronous region. Some additional comments here could benefit the paper. Is the oscillation frequency of JT/WC constant throughout simulations, and throughout the synchronous zone, or does it vary?

▷ Response: Thanks. The oscillation frequency of JR/WC is constant throughout simulations.

▶ Reviewer: The authors use the coefficient of variation (standard deviation over the mean, across simulations instances) as a "marker of criticality". While I understand the rationale behind this choice, I would like to see better justifications or literature references detailing why CV over simulation instances would be accurate to detect criticality. For example, you could also also compute the CV over time for each simulation (standard deviation of the synchronization parameter over time), and then average that across simulations. This would give a measure of the variability of synchronization within simulation instances, which could also be an interesting metric and arguably also an apt marker of criticality.

▷ Response: The authors are grateful for your detailed vision. To investigate the SOPT, we compute the coefficient of variation (CV) of synchronization value against the control parameter (coupling coefficient). The sharp increase in the CV of the synchronization value across the coupling coefficient at the critical point is a key indicator of critical slowing down [11]. This phenomenon is particularly relevant in the study of SOPT and the behavior of complex systems near their critical points [7]. Indeed, the CV is a measure of the relative variability of a data series around its mean, and its increase at the critical point suggests that the variability of the synchronization value is significantly affected by the coupling coefficient at this point. This increase in CV can be interpreted as a sign that the system behavior is becoming more sensitive to changes in the coupling coefficient, indicating that the system is nearing a critical point and its dynamics undergo a qualitative change (highlighted text in the "Measure of criticality" subsection in the new version of the manuscript).

▶ Reviewer: Looking at the simulated activity, it is clear that synchronous oscillations in the WC model are much, much slower than in the JT model. The author's chosen duration of 200s for the simulation is then apt to capture many cycles of JT oscillations, but very few cycles for the WC model. Please consider running longer simulations for the WC model so that the number of cycles is at least

comparable to JT, or adapt the WC model so that its temporal oscillations are closer in frequency to JT, so that the computation of their synchronization parameters relies on a similar number of cycles.

▷ Response: Thanks for your careful consideration. It is notable that in both models, the system achieved equilibrium, ensuring that the pattern remains unchanged. Two arbitrary simulations for $K = 0.075, 0.15$ in WC model are presented in figures 1 and 2 respectively, for time simulations of 200 and 1000 seconds. It is evident that the pattern remains unchanged.

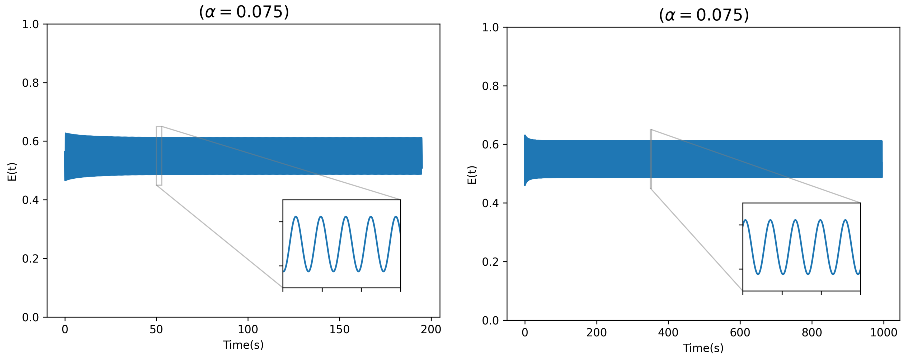

Fig. 1: The time series of the mean network activity derived from the WC model during 200 seconds (left) and 1000 seconds (right) simulations at $\mathcal{K} = 0.075$

.

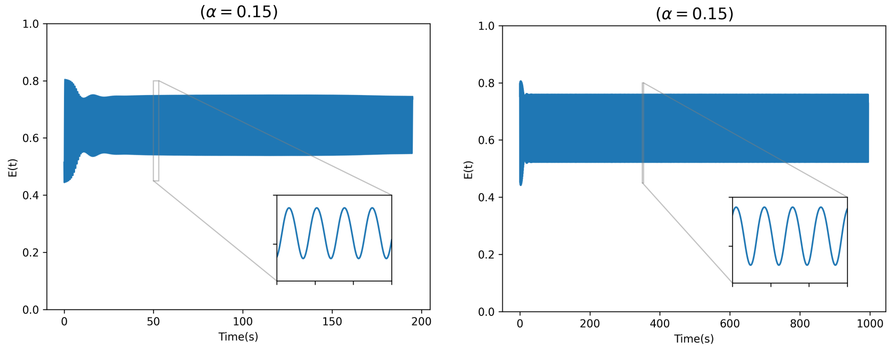

Fig. 2: The time series of the mean network activity derived from the WC model during 200 seconds (left) and 1000 seconds simulations (right) at $\mathcal{K} = 0.15$

.

▶ Reviewer: Consider running additional simulations for WC model for values of the control parameter near the expected transition points (e.g. try values 0.0625, 0.1875 and nearby). Your steps of coupling strengths are quite large for the WC model, and might be stepping over a transition.

▷ Response: We agree with this and have simulated for values 0.05625, 0.0625, 0.6875, and also 0.18125, 0.1875, 0.19375. The new Kuramoto order parameter value according to the coupling coefficient in the WC model is presented in figure 3. It shows that our results in the previous version of the manuscript remain unchanged. Due to these new results, figures 2, 5 have been updated in the new version of the manuscript.

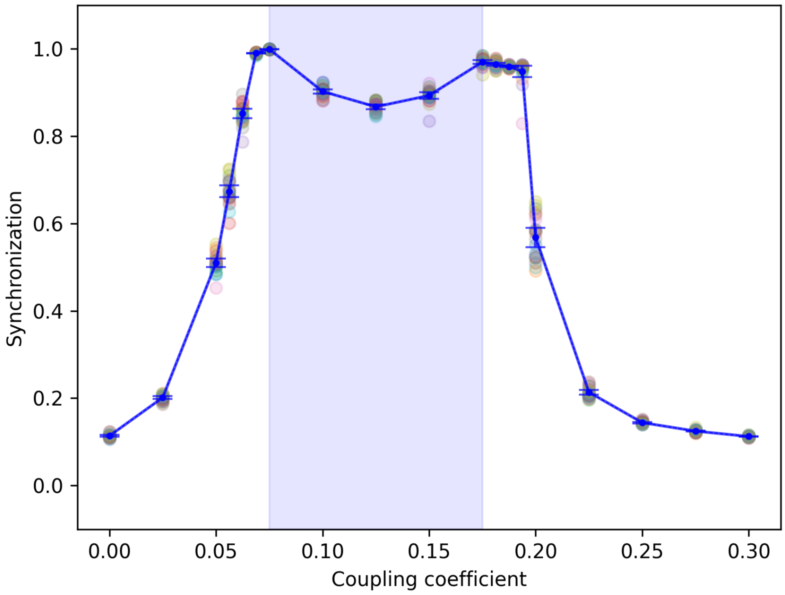

Fig. 3: The KOP value according to the coupling coeficient in WC model

.

▶ Reviewer: Some figures lack clear axis labels. While the authors do provide information in the text and captions that clarifies the figures content, the figures themselves should also be readily understandable. For example, the x-axis of simulation figures should directly show the temporal duration; the units and significance of the DFA figure axes should not (only) use mathematical symbols, but also an explicit description (e.g. time window (seconds)).

▷ Response: Thanks. These figures have been improved.

▶ Reviewer: I do not find any links to github or similar repositories for access to the code used in this work. Please provide links to static repositories of your code upon revision.

▷ Response: Thanks. We have added the ”Data Availability” section.

Comments from Reviewer 3:

▶ Reviewer: First, the manuscript is mostly detached from what has been done in the past. A quick search returns multiple papers on synchronization of WC oscillators (see e.g. DOIs 10.1109/72.501714, 10.1142/S0218127403006406) and some on JR system (10.1016/j.ifacol.2017.08.2577). Also, the whole second paragraph of the introduction is not clear: is this about neural network in the neuroscience sense or in the general sense? If it's about networks of real neurons, it's not clear how other applications mentioned in this para are related.

▷ Response: Thanks for your comment. These references have been added.

Several statements we made were in the second paragraph of the introduction more ambiguous than intended, and we have adjusted the text to be clearer. This paragraph has a general sense of the importance of synchronization in biological systems and other fields.

▶ Reviewer: Second, the language in the paper is convoluted or incorrect at many spots. In particular, many times I met "which" in the text, it left me guessing what it refers to (e.g. lines 5 in the abstract, lines 89, 231). Line 17 says "models depend on their outputs" – In what sense? Line 243 "the increasing trend of the network by increasing coupling strength" does not make sense. It's a milder disconnect, but still "synchronization" and "asynchronization" are used in parallel, whereas synchronization is mostly a process, and "desynchronization" is mostly used for the opposite of it. If the authors mean states, then it's more accepted to say "synchronous" or "asynchronous" states. The notions are not properly defined and introduced. For example, what is "power-law behavior" mentioned on line 55? What is Wats-Strogatz topology (line 137). Why the paper that introduces this notion is not referenced in that same sentence? Additionally, I'm completely lost on the probability of rewiring – is the network plastic and connections may be formed and disappear in time? This needs to be introduced in more details, and a picture may help here. The inequalities on the parameters of topology are confusing for me, but it's also my understanding that the authors did not do any parameter search for the power law behavior. Was the model simulated only for one set of parameters? Then their justification should be very through especially as the result is that the networks cannot display power-law dynamics. The notation for the JR system is not optimal as the choice of the same letter for the firing rate and the potential (line 114)

impedes it comprehension. Furthermore, it's not clear why the difference $y_1 - y_2$ represents output of the model and postsynaptic membrane potential of pyramidal neurons, whereas y_0 was attributed to these neurons. Again, milder issue, but also contributes to impeding understanding: the variables in Eq (4) are listed not in a logical order. Maybe I don't grasp the logic of it.

▷ Response: We apologize for this error. Our manuscript has been reviewed by a colleague and revised to improve readability.

- These sentences have been rephrase as follows: (line 5 in the Abstract): Despite extensive research on these models over the past several decades, their potential of manifesting second-order phase transitions (SOPT) and criticality has not been sufficiently acknowledged.
- Line 89: Table 1 shows the values of parameters with their interpretations. These values can produce oscillatory behavior in the WC model.
- Line 231: When the coupling coefficient is set to 1.25, the network's synchronization significantly increases, indicating a potential disorder or instability in the network's function.
- Line 17: These models depending on their output (voltage or rate) are classified into two voltage- and activity-based models.
- Line 243: Indeed, Fig 2(B) shows a local minimum in the KOP in which by increasing coupling strength, the synchronization value decreases.
- The "desynchronization" expression has been replaced by "asynchronization". Moreover, expressions of synchronous or asynchronous states have been edited.
- Line 55: Power-law distributions are crucial not only in neuronal systems but also in other vital apparatuses for living organisms. Scientists have been trying to observe power-law fluctuations in different temporal data and investigating their origins. By increasing the volume of this data, we can gain a better understanding of complex systems through empirical observations. Power-law behavior in various variables appears in critical dynamics. For example, the size and duration distributions of neuronal avalanches and EEG cascade obey a power-law. Moreover, long-range temporal correlations have been revealed in the amplitude envelopes of neural oscillations. The conceptual appeal of the power law distribution indicates that the system is operating near a critical point and is poising in

a boundary between different types of dynamics that networks show the optimal performance of a cortical function.

- Line 137: The relevant reference has been added. Moreover, we have explained more detail about rewiring (highlighted text in the ”Network structure” section in the new version of the manuscript).
- We have added these limitations in the ”Discussion and Conclusion” section: It is notable that different parameter sets and different topologies have not been discussed, which implies the limitation of our research. These results do not apply to the WC or JR models in general, but with a specific parameter set and on a specific network topology. Different topologies, or different parameter sets, are likely to exhibit entirely different behaviors. Moreover, we emphasis that these analysis does not exclude that transitions could be generated also by varying other model parameters, or with different topologies.
- The model features a population of pyramidal neurons (central part of figure 1.a.) that receive excitatory and inhibitory feedback from local inter-neurons and excitatory input from neighboring cortical units and sub-cortical structures like the thalamus. Actually, the excitatory feedback must be considered as coming from both local pyramidal neurons and genuine excitatory interneurons like spiny stellate cells.

  Figure 1b. is a translation of figure 4.a in the language of system theory. It represents the mathematical operations performed inside such a cortical unit. The excitatory input is represented by an arbitrary average firing rate $p(t)$ which can be random (accounting for a nonspecific background activity) or deterministic, accounting for some specific activity in other cortical units. The three families pyramidal neurons, excitatory and inhibitory inter-neurons , and synaptic interactions between them are modeled by different systems.

  The Post-synaptic systems P_i; $i = 1; 2; 3$ (labeled $h_e(t)$ or $h_i(t)$ in the figure) convert the average firing rate describing the input to a population into an average excitatory or inhibitory post-synaptic potential (EPSP or IPSP). From the signal processing standpoint, they are linear stationary systems that are described either by a convolution with an impulse response function or, equivalently, by a second-order linear differential equation. They have been proposed by van Rotterdam in order to

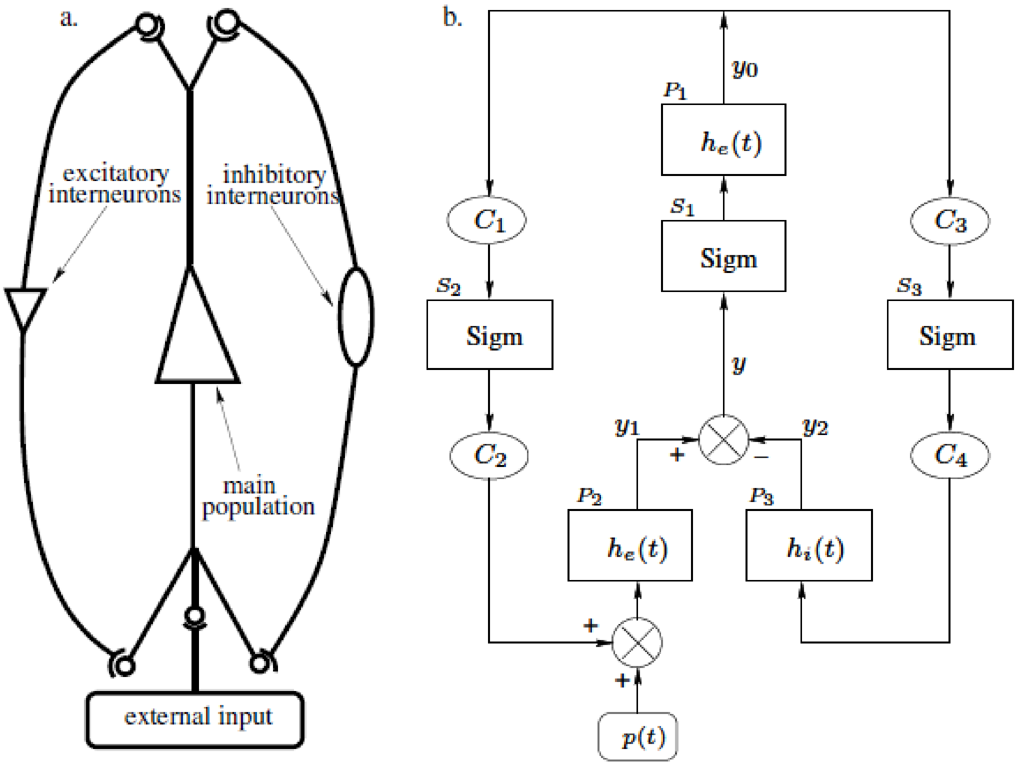

Fig. 4: a) Neural mass model of a cortical unit: it features a population of pyramidal cells interacting with two populations of inter-neurons, one excitatory (left branch) and the other inhibitory (right branch). b) Block representation of a unit. The h boxes simulate synapses between the neurons populations. Sigm boxes simulate cell bodies of neurons by transforming the membrane potential of a population into an output firing rate. The constants C_i model the strength of the synaptic connections between populations.

reproduce well the characteristics of real EPSPs and IPSPs. The impulse response function is of the form:

$$\mathrm{h(t)} = \begin{cases} \alpha\beta t e^{-\beta t} & t \geq 0 \\ 0 & t < 0 \end{cases}$$

In other words, if $x(t)$ is the input to the system, its output $y(t)$ is the convolution product $h * x(t)$. The constants α and β are different in the excitatory and inhibitory cases. α expressed in millivolts, determines the maximal amplitude of the post-synaptic potentials, β expressed in s^{-1}, lumps

together characteristic delays of the synaptic transmission, i.e. the time constant of the membrane and the different delays in the dendritic tree.

The corresponding differential equation is:

$$\ddot{y}(t) = \alpha\beta x(t) - 2\beta\dot{y}(t) - \beta^2 y(t) \tag{1}$$

In the excitatory (respectively inhibitory) case we have α = A, β = a (respectively α = B, β = b). This second-order differential equation can be conveniently rewritten as a system of two first-order equations:

$$\begin{cases} \dot{y}(t) = z(t) \\ \dot{z}(t) = \alpha\beta x(t) - 2\alpha z(t) - \alpha^2 y(t) \end{cases} \tag{2}$$

There are three main variables in the model, the outputs of the three post-synaptic boxes noted y_0, y_1 and y_2 (see figure 1.b); we also consider their derivatives $\dot{y}_0$, $\dot{y}_1$, $\dot{y}_2$ noted y_3, y_4 and y_5, respectively. If we write two equations similar to equation 2 for each postsynaptic system we obtain a system of 6 first-order differential equations that describes Jansen's neural mass model:

$$\begin{aligned} \dot{y}_0(t) &= y_3(t) \\ \dot{y}_3(t) &= Aa\mathcal{S}(y_1(t) - y_2(t)) - 2ay_3(t) - a^2 y_0(t) \\ \dot{y}_1(t) &= y_4(t) \\ \dot{y}_4(t) &= Aa\left\{p(t) + C_2\mathcal{S}[C_1 y_0(t)]\right\} - 2ay_4(t) - a^2 y_1(t) \\ \dot{y}_2(t) &= y_5(t) \\ \dot{y}_5(t) &= BbC_4\mathcal{S}(C_3 y_0) - 2by_5(t) - b^2 y_2(t) \end{aligned} \tag{3}$$

The full explanation of JR model is gathered from the following references:

Grimbert, F., Faugeras, O. (2006). Bifurcation analysis of Jansen's neural mass model. Neural computation, 18(12), 3052-3068.

We have tried to explain better in the "Jansen-Rit model" subsection to be clear and in some cases just to mention the references.

▶ Reviewer: Third, I think that the model is insufficiently justified and illustrated. A diagram that shows multiple WC and JR oscillators and how they're connected would be much more useful either

as a replacement of Fig 1, or as a part of it. I also don't understand why the nodes interact through excitatory connections only (lines 93-94; a reference is not provided). By contrast, I think that a network where two excitatory populations are connected through interneurons, comprising a bistable circuit, is very common.

If the parameter choice for the WC system is justified by mentioning oscillatory dynamics, there is no such a justification for the JR system. Moreover, the system is quite complex and may be capable of producing dynamics more complex than periodic oscillations. Such issues should be discussed as a part of parameter calibration. Another issue with the complexity of JR system is that it can be connected in many different ways, and the choice made in the manuscript need yet more solid justification.

I am also missing what desynchronizes the oscillators. If they are identical, they have the same natural frequencies, and, therefore, require very weak interaction to synchronize.

▷ Response: Thanks for pointing these out.

- Figure 1 has been edited to be more clear.
- Line 93: The related reference has been added.
- Various methods exist for modeling the brain, including mesoscale or neural mass models. These models focus on interactions between distinct brain regions rather than individual neurons. Given that interactions between regions are primarily excitatory, with inhibitory interactions being localized, mesoscale models have been designed to account for these excitatory interactions exclusively [10].
- According to Van Rotterdam's work, the parameters A, B, a, and b have been determined so that alpha activity can be produced by the system while respecting some basic properties of real post-synaptic potentials [9]. v_0, v_{max}, and r $= 0.56mV^{-1}$ are derived from the experimental studies of Freeman [2]. $C_i(i = 1, ...4)$ are proportional to the average number of synapses between populations. Jansen and Rit reduced these quantities to a single parameter C based on a neuroanatomical study [3]

that used synaptic counting to estimate these quantities:

$$C_1 = C$$

$$C_2 = 0.8C$$

$$C_3 = 0.25C$$

$$C_4 = 0.25C$$

Using these values, Jansen and Rit varied C to observe alpha-like activity and obtained it for C = 135 [5].

- In some research areas, desynchronization might be a key interest. For example, in the study of complex systems, desynchronization can lead to the emergence of new states and behaviors that are not observed in synchronized systems. The interest in desynchronization can vary widely depending on the specific goals and applications of the research. This research discusses the importance of synchronization in two models. What desynchronizes the oscillators is out of scope and we suggest this in future works.

► Reviewer: Finally, I'm not sure if the analysis is sound. The text says that CV only "may be" a marker of criticality (line 175). This undermines the validity of the results.

▷ Response: Several statements that we made were more ambiguous than intended, and we have adjusted the text to be clearer. CV is a marker of criticality, but it is not sufficient to prove criticality.

Some minor comments:

► Reviewer: Lines 162 and 163 introduces a transition between synchronous and asynchronous states as a transition to chaos. Chaos is a very particular term. Is it really clear that asynchronous state is chaotic?

▷ Response: The authors agree with your comment. So, we have removed this term.

► Reviewer: Line 191 says that we have $2N_T$ segments, and I don't understand where the 2 is coming from.

▷ Response: Several statements that we made were more ambiguous than intended, and we have adjusted the text to be clearer. This situation arises when the total length of the signal (N) is not exactly divisible by the segment length (T), leading to a remainder that cannot be accommodated within the defined segment length. This can result in the last segment being shorter than the others, potentially missing valuable data or introducing inaccuracies in the analysis. To address this issue, a common strategy is to divide the signal from its end, ensuring that each segment is of equal length and that no data is lost. This approach involves creating additional segments at the beginning of the signal, effectively padding the signal with extra data points. By doing so, the total number of segments becomes $2N_T$, where N is the original signal length and T is the segment length. This method ensures that every segment is of equal length, maintaining consistency and integrity in the analysis or processing of the signal (highlighted text in the subsection of ”Measure of criticality”).

Please note:

The authors believe that the comments and suggestions made by the referees have improved the paper. We look forward to hearing from you in due time regarding our submission and to respond to any further questions and comments you may have.

Sincerely,

Yousef Jamali

References

1. John M Beggs and Dietmar Plenz. Neuronal avalanches in neocortical circuits. *Journal of neuroscience*, 23(35):11167–11177, 2003.
2. Walter J Freeman. Simulation of chaotic eeg patterns with a dynamic model of the olfactory system. *Biological cybernetics*, 56(2):139–150, 1987.
3. Laurence Garey. Cortex: Statistics and geometry of neuronal connectivity, 2nd edn. by v. braitenberg and a. schüz.(pp. xiii+ 249; 90 figures; isbn 3 540 63816 4). berlin: Springer. 1998. *The Journal of Anatomy*, 194(1):153–157, 1999.
4. Clayton Haldeman and John M Beggs. Critical branching captures activity in living neural networks and maximizes the number of metastable states. *Physical review letters*, 94(5):058101, 2005.
5. Ben H Jansen and Vincent G Rit. Electroencephalogram and visual evoked potential generation in a mathematical model of coupled cortical columns. *Biological cybernetics*, 73(4):357–366, 1995.
6. Mahsa Khoshkhou and Afshin Montakhab. Spike-timing-dependent plasticity with axonal delay tunes networks of izhikevich neurons to the edge of synchronization transition with scale-free avalanches. *Frontiers in systems neuroscience*, 13:73, 2019.
7. Marten Scheffer, Jordi Bascompte, William A Brock, Victor Brovkin, Stephen R Carpenter, Vasilis Dakos, Hermann Held, Egbert H Van Nes, Max Rietkerk, and George Sugihara. Early-warning signals for critical transitions. *Nature*, 461(7260):53–59, 2009.
8. H. Eugene Stanley and Victor K. Wong. Introduction to phase transitions and critical phenomena. *American Journal of Physics*, 40(6), 1972.
9. 3A van Rotterdam, FH Lopes Da Silva, J Van den Ende, MA Viergever, and AJ Hermans. A model of the spatial-temporal characteristics of the alpha rhythm. *Bulletin of mathematical biology*, 44(2):283–305, 1982.
10. Hugh R Wilson and Jack D Cowan. Excitatory and inhibitory interactions in localized populations of model neurons. *Biophysical journal*, 12(1):1–24, 1972.
11. Hongdian Yang, Woodrow L Shew, Rajarshi Roy, and Dietmar Plenz. Maximal variability of phase synchrony in cortical networks with neuronal avalanches. *Journal of neuroscience*, 32(3):1061–1072, 2012.
