# Supplementary material for: Criticality and partial synchronization analysis in Wilson-Cowan and Jansen-Rit neural mass models
Source: PLoS One. 2024 Jul 3;19(7):e0292910. doi: 10.1371/journal.pone.0292910 (PMC11221676; doi:10.1371/journal.pone.0292910)
Supplement: Attachment — Submitted filename: Lorenzi_rev_PONE-D-23-31916.docx [file pone.0292910.s002.docx]

The paper is well written and report the results of the comparison between JR and WC model in a concise way. The evaluation of computational model performance has a crucial importance in the field of computational neuroscience. For what concerning Editor’s comments, in my opinion, the authors update the manuscript in a proper manner.

Here below I share my comments concerning minor modifications which could improve the manuscript clarity.

**Introduction:**

In the introduction the background is explain in a clear and concise form. However, some point it lacks practical examples, such as in line 37 and in line 41. I appreciated the intention to make an extensive overview of mean field applications in different fields, but I would add a practical example on how these implications can be explored by analyzing the mean field synchronization.

**Methods:**

All the parameters are explicit indicated in the description of WC equation but I suggest also adding the unit of measurement of each parameter to make equation clearer.

Regarding the choice of using excitatory connection, this is true in the cerebrum, but in the cerebellum the connection between cerebellar cortex and deep cerebellar nuclei are driven by inhibitory coupling, therefore I suggest changing the sentence “as is true of the brain”.

As far as I understood, parameters in Table 1 for WC model are derived from a previous work of the authors but no information are provided for parameters in Table 2 for JR model. I recommend adding the reference if authors taken them from literature, or explaining how they are derived in this work.

The method used to build up the network is clearly reported. I would like to request to disentangle the meaning of adjacency and coupling parameters and how they are determined. Are they derived from the structural connectivity mentioned in the manuscript (lines 117-120)?

Regarding the network structure I would suggest adding some further details about what small-world topologies are. Furthermore, I recommend adding a comment about the reason why p is the most challenging parameters to be estimated.

In “Measure of criticality” section, the context is well introduced. I would add some examples regarding the assessed measures of criticality mentioned in line 169.

In “Simulations” section, simulations set up doesn’t result completely clear to me. Why are simulations run 20 times? And in the 20 repetitions for each simulation, which are the changing parameters? Furthermore, I recommend adding an explanation about how the coupling strength range was set up for the simulations.

**Results:**

Results are promising and prompt for applying SOPT analysis in brain modeling fields.

Sometimes the authors referred the coupling parameter as “coupling parameter” or “K”. This is a bit confusing, and I would suggest using “K” since it was already defined in methods part.

Furthermore, I recommend changing the title of heatmaps in figure 3 and 4 replacing alpha with K to be consistent with the rest of the manuscript.

**Discussions:**

Discussions are presented in a concise way. I would suggest adding the authors point of view supported by the manuscript results on when JR is more suitable than WC for brain dynamics modeling and the vice versa.

**Specific Comments:**

- Line 54: I would replace “optimal” with “physiological”.
- Line 77, as many mean field approximations exists (such as Lorentzian base, heuristic TF based….), I would add a reference for the mean filed rational applied to WC model.
- Line 132, if the “connections” are referred to structural connectome of neurons/brain regions, I would suggest using “biological” instead of physical.
- Line 137 The Reference for Watts-Strogatz topology is missed.
- Line 180: “long-range temporal correlations” is already defined as LRTC; therefore I would replace it with the acronym.
- Line 314 typo: Delete the “.”
- Figure 1: I suggest adding the title to the panels also in the figure to make it more clear at a first look. I would swap Panel A and Panel B consistently to the following figures where A is JR and B WC.
- Figure 4. Put [Hz] next to Rate as Activity[mV] in figure 3.
